# Impact of ChatGPT on medical chatbots as a disruptive technology

**DOI:** 10.3389/frai.2023.1166014

**Published:** 2023-04-05

**Authors:** James C. L. Chow, Leslie Sanders, Kay Li

**Affiliations:** ^1^Radiation Medicine Program, Princess Margaret Cancer Centre, University Health Network, Toronto, ON, Canada; ^2^Department of Radiation Oncology, University of Toronto, Toronto, ON, Canada; ^3^Department of Humanities, York University, Toronto, ON, Canada; ^4^Department of English, University of Toronto, Toronto, ON, Canada

**Keywords:** ChatGPT, medical chatbot, artificial intelligence, disruptive technology, healthcare, nature language processing, knowledge base, big data

## 1. Introduction

Sweeping changes in artificial intelligence (AI) have been brought about in recent years, resulting in remarkable progress taking a number of forms, such as AI chatbots. ChatGPT (Chat Generative Pre-trained Transformer) is a language model for dialogue. This chatbot, developed by Open AI, was released in prototype form on November 30, 2022 (ChatGPT, 2023). Since then, ChatGPT has attracted numerous users from various fields, because it can provide detailed answers and humanlike responses to almost any question. ChatGPT is reputed to be serving various medical functions, ranging from uses in medical writing and documentation to medical education. Recently, ChatGPT has been reported to be capable of passing the gold-standard US medical exam, suggesting that is has potentially significant applications in the field of medicine (Kung et al., 2023).

However, it is questionable whether ChatGPT can consistently provide reliable health information for patients or healthcare providers interacting with it. A reliable medical chatbot could constitute a seamless interface to information for both patients and healthcare providers. As a patient-oriented tool, it would allow users to obtain disease-related information or book medical appointments (Bates, 2019; Khadija et al., 2021). Simultaneously, it could serve healthcare professionals by providing essential information relating to their work, such as medical protocols for treatment of common and rare diseases or hospital policies (Bates, 2019; Gupta et al., 2021; Wan, 2021).

Unlike a specific medical chatbot, ChatGPT has not been trained on a finely-tuned dataset created by medical professionals (Sallam, 2023). This raises concerns, as patients may initially turn to ChatGPT for assistance. While this tool has the potential to educate and expedite care, there is also a risk that it may provide inaccurate diagnoses or recommendations (Cascella et al., 2023). Furthermore, the chatbot's machine learning and data search algorithms are still in the prototype phase, and the development of related ethical policies and regulations is ongoing (Liebrenz et al., 2023).

As researchers studying the design and creation of medical chatbots, we expect that ChatGPT will be able to evolve into a reliable and practical medical chatbot. Here, we would like to explore some obstacles to the achievement of this goal and potential solutions to them, by considering ChatGPT as a disruptive technology.

## 2. ChatGPT as a disruptive technology

Disruptive technologies often begin as niche solutions or products with limited initial market appeal. Over time, they gain acceptance and transform the industry or market they are a part of (Kostoff et al., 2004). A prime example is the digital camera, which eliminated the need for film and traditional film processing. Before digital cameras, film cameras dominated the photography market. However, digital cameras disrupted this market by offering a more convenient and cost-effective alternative. ChatGPT is also a disruptive technology with the potential to fundamentally change how we interact with technology and perhaps to revolutionize the way medical professionals engage with patients. If ChatGPT can function as a professionally trained medical chatbot, it may be able to operate more quickly than existing medical chatbots, draw on a larger database, reduce medical errors, and assist doctors in improving their performance.^1^ A concern with any disruptive technology is that, while it may be innovative, timely, and impactful, it also carries risks and it may not immediately integrate well with complex, professional applications, such as those that are relevant in medicine.

Currently, several obstacles hinder ChatGPT from functioning fully as a medical chatbot. For instance, its database may not be entirely up to date; the current knowledge cutoff is September 2021. Additionally, medical information sourced from the internet might not be entirely accurate, posing a risk of providing misinformed answers.^2^ Numerous ethical concerns exist, including patient safety, privacy, data content, and cybersecurity (Xu et al., 2021; Parviainen and Rantala, 2022). Caution is necessary for clinical applications, and medical professionals are working to verify and fine-tune the chatbot. User feedback influences the chatbot's training, but users may not understand the interaction model, making adoption more difficult. Shifting the culture of medical service from human-to-human to machine-to-human interactions will take time. Finally, rapid AI advancements will continuously modify the ethical framework (Parviainen and Rantala, 2022). This process is expected to be lengthy and time-consuming for various stakeholders, such as medical service providers, AI developers, and users.

We must admit that the chatbot is extremely popular, as it is very user-friendly, and patients as users may focus more on the benefits of convenience and efficiency, rather than the reliability and accuracy of the tool (Chin et al., 2023). ChatGPT also presents an air of authority and so sounds rather trustworthy. This is particularly noteworthy during the period of the recent pandemic, during which medical resources have been limited, and virtual chats have become quite the norm. At its present stage of development, caution probably needs to be exercised by both medical service providers and patients in making serious and ethical use of the medication information provided by ChatGPT, given its nature as a disruptive technology. Medical service providers also need to acquire a detailed understanding from AI developers of the data and conversational flow algorithm underlying the AI chatbot.

Nevertheless, although ChatGPT is currently still imperfect as a humanlike medical chatbot, we believe that it is bound to change healthcare systems in the near future. Below, we explore some obstacles to that goal and discuss potential solutions to each obstacle.

## 3. Some obstacles on the path to ChatGPT becoming a medical chatbot

### 3.1. Current medical chatbots vs. ChatGPT

ChatGPT may not be as accurate as medical chatbots developed by dedicated medical professionals. Medical chatbots that have been built over the years often use AI features like natural language processing (Hirschberg and Manning, 2015) and focus on specific tasks, such as answering users' inquiries on various medical topics (Kovacek and Chow, 2021; Rebelo et al., 2022). Chatbot creators can build the underlying software, link it to a maintained database, and easily modify the conversational flow and data. In contrast, ChatGPT was not trained by a specialized team of medical professionals (Chow, 2021; Siddique and Chow, 2021). Its strength lies in its natural language processing (NLP) capabilities, based on the Generative Pre-trained Transformer (GPT-3.5) form of AI. This allows ChatGPT to extract information from unstructured data sources, such as electronic health records (EHRs), identify patterns and recurrences, such as particular symptoms, and generate diagnostic reports.^3^ This can potentially reduce the workload of frontline health workers during routine medical checks and could help to alleviate shortages of healthcare workers. Recently, ChatGPT has been upgraded with new features and improvements. The new language model (GPT-4), which is linked to Bing Chat, can process up to 25,000 words and boasts advanced capabilities in creativity and visual input, as well as longer contextual memory, compared to its predecessor, GPT-3.5. According to internal testing, ChatGPT now reportedly provides 40% more factual responses, making it a safer and more accurate tool for tasks such as music composition, screenwriting, and technical writing.^4^

Traditional medical chatbots use AI and natural language processing to predict user intent and provide appropriate responses (Chow et al., 2023). They are continuously improved through user feedback and performance data. These processes are controlled by chatbot creators using a well-maintained, human-designed database. However, ChatGPT, as a disruptive technology, draws information from the internet, making the accuracy and currency of the medical information it supplies questionable and sometimes uncontrollable. Although this approach saves time and effort in database preparation, ChatGPT requires careful training from medical professionals, as it may be trained by any user, which can lead to inaccurate information. Therefore, it is crucial to test and evaluate ChatGPT's performance, as its responses may be unpredictable and dependent on the data used for training. Development of a robust quality assurance system and a systematic approach to monitoring of database updates and maintenance can help to ensure the accuracy and precision of the information provided by ChatGPT. In addition, the chatbot creator can partner with a team of medical experts to review and validate the dataset used to train ChatGPT, so that a custom training dataset can be built to improve the accuracy and relevance of ChatGPT's medical knowledge. Continuous monitoring and improvement are also necessary to monitor the performance of ChatGPT as a medical chatbot, to ensure that it can remain current and relevant through regular updating of the training data with new and accurate information.

### 3.2. Human doctors vs. AI doctors

The current medical system relies on certified professionals to provide reliable services to patients. These professionals need to maintain their certifications, ensuring quality care. However, AI-based chatbots such as ChatGPT do not undergo any similar verification process, raising ethical concerns. AI chatbots could provide a quick solution to the high demand for medical care during situations like pandemics. The fact that ChatGPT has passed the Medical Boards examination may increase public acceptance and trust in AI systems in the healthcare domain. As people become more familiar with AI technologies, they might be more open to incorporating AI-based tools into their healthcare routines. This increased acceptance may lead to further integration of AI in the medical field, enhancing the efficiency and effectiveness of healthcare services. However, it is important to remember that passing the Medical Boards examination does not necessarily make ChatGPT a complete substitute for human medical professionals. Practical experience, empathy, and interpersonal skills are essential components of healthcare that AI systems do not easily replicate. Additionally, ChatGPT's performance on the examination may not fully represent its ability to handle complex and nuanced medical situations in real-world settings.

To address the challenges of using ChatGPT in medicine, medical professional organizations should consider establishing suitable frameworks to monitor and assess the quality of ChatGPT for applications in healthcare. This will involve the provision of clear guidelines for users on how to use ChatGPT correctly and guidance for service providers on safely implementing ChatGPT as a medical chatbot. A major consideration should involve setting parameters for the safe usage of ChatGPT. For example, its functions could be limited to particular areas where ChatGPT has demonstrated accuracy, such as diagnosis, education, and healthcare. Through implementation of these measures, ChatGPT could become an invaluable asset to the medical profession.

### 3.3. Ethical concerns

Since the pandemic period, medical chatbots have been rapidly developed as conversational agents for use by patients, thus accelerating the development and deployment by medical ethicists of corresponding ethical frameworks for this disruptive technology. To date, many legal and ethical challenges have already emerged regarding medical chatbots that need to be addressed and dealt with (Liebrenz et al., 2023). These include the data content of the chatbot, cybersecurity, data use, privacy and integration, patient safety, and trust and transparency between all participants. The construction of such ethical frameworks will take time because it is dependent on patients' feedback and robust updating of the chatbot itself. It also involves a great deal of negotiation among various stakeholders, for example, concerning patient data and their ownership. The present progress in the deployment of such ethical frameworks cannot keep pace with the rapid advancement of ChatGPT as a medical chatbot. This will exert an increasing amount of pressure on medical professionals when they want to implement this type of disruptive technology in the medical system within such a short period of time.

Continued systematic research into the ethical implications of ChatGPT for its users is necessary, and international collaborations should be pursued to establish a global standard for ethics surrounding the use of ChatGPT as a medical chatbot. In order to address issues related to data content, cybersecurity, privacy, and integration, various stakeholders (including medical professionals, patient and hospital representatives, and computer security experts) should convene to establish policies regarding patient data ownership and security. Adequate protection of patient data must be implemented as a standard regulation to ensure patient privacy when using the chatbot on the Internet of Things. In order to promote patient safety, trust, and transparency, ethical guidelines and protocols should be developed and put in place to govern the appropriate use of AI-generated medical advice. Users should receive education on the limitations of and potential risks associated with using ChatGPT as a medical chatbot. We also recommend that users verify any critical information with healthcare professionals before making any decisions related to their health. Medical ethics must serve as guiding principles in shaping the ethical framework for ChatGPT as a provider of medical information and medical “practitioner”.

## 4. Summary

While ChatGPT has the potential, as a disruptive technology, to improve access to healthcare services, there are also concerns relating to its use as a medical chatbot. One concern is the accuracy and reliability of the medical information provided by ChatGPT, as it is not a licensed medical professional and may not have access to up-to-date medical knowledge. Additionally, there are concerns about the transparency of the chatbot model and the ethics of making use of user information, as well as the potential for biases in the data used to train ChatGPT's algorithms. As such, it is important to carefully consider the potential risks and benefits of using ChatGPT as a medical chatbot, and to ensure that appropriate safeguards are put in place to address these concerns. As we believe that ChatGPT will be further developed into a humanlike medical chatbot in the future, we urge relevant stakeholders to continue studying and improving the chatbot. Moreover, we urge them to engage in serious examination of the obstacles to achieving this goal as soon as possible, in order that related standards for quality assurance systems and regulations can be established, so as to keep pace with the challenges posed by this disruptive technology.

## Author contributions

JC and KL wrote the article. JC, KL, and LS revised the article. All authors contributed to the article and approved the submitted version.

## Footnotes

^1^ https://www.forbes.com/sites/robertpearl/2023/02/13/5-ways-chatgpt-will-change-healthcare-forever-for-better/?sh=8b101f07bfc

^2^ https://medium.com/@asarav/the-limitations-of-chat-gpt-8b73f5859bb4

^3^ https://healthydebate.ca/2023/02/topic/future-medicine-chatgpt/

^4^ https://www.digitaltrends.com/computing/chatgpt-4-everything-we-know-so-far/
